# Change in the Characteristics of Patients Qualified for Hearing Aids over the Last 25 Years in Poland

**DOI:** 10.3390/jcm12175625

**Published:** 2023-08-29

**Authors:** Karolina Dżaman, Marlena Ziemska-Gorczyca, Ireneusz Kantor

**Affiliations:** Department of Otolaryngology, Centre of Postgraduate Medical Education, Marymoncka 99/103, 01-813 Warsaw, Poland; kfrydel@poczta.onet.pl (K.D.); ireneusz.kantor@gmail.com (I.K.)

**Keywords:** hearing loss, hearing aids, audiology

## Abstract

Hearing loss is one of the most common causes of disability worldwide. The aim of the study was to compare the demographic structure and the results of hearing tests in people qualified for hearing aids over the last 25 years. The material covered 1246 patients qualified for hearing aids in the years 1996–2001 and 2016–2021. Patients were divided into two groups according to the time of qualifying for hearing aids. Group 1 (G1) consisted of 759 people qualified in the years 1996–2001, and Group 2 (G2) comprised 487 people qualified in the years 2016–2021. Statistical analysis was performed on the results of pure tone threshold audiometry and the demographic structure in both groups. Patients in G1 had statistically significantly elevated hearing thresholds (HT) in the air conduction range at frequencies from 2000 to 8000 Hz in relation to G2 patients. The opposite situation was observed for the bone conduction threshold. G2 patients had significantly elevated bone conduction HT at frequencies from 250 to 1000 Hz compared to G1 patients. The age structure in both groups was similar; however, the gender distribution was statistically significantly different. In G1 women accounted for 40%, and in G2, they became the dominant gender (53%). Over the last twenty years, there has been a change in the structure of patients qualified for hearing aids. Although the age has remained similar, today, patients decide to use hearing aids at an earlier stage of hearing loss than 25 years ago. Modern women began to use hearing aids much more often.

## 1. Introduction

According to data published by the World Health Organization (WHO), it is estimated that in 2019, around 1.57 billion people worldwide had a hearing impairment, and 62.1% of them were over the age of 50 [1]. In the U.S. alone, approximately 37.5 million (15%) of adults over the age of 18 report hearing impairment [2]. This makes age-related hearing loss the third largest source of disability globally in 2019, dominating as the leading cause of disability among people over the age of 70 [3].

The basic consequence of disability, which is hearing loss, and in particular sensorineural hearing loss, is a reduced ability to recognize speech (speech in noise), a problem with communication and an increased risk of isolation, which results in a significant decrease in the quality of life.

In people with hearing loss who cannot be qualified for surgical treatment, the only way to alleviate the effects of hearing impairment and improve speech understanding is the use of various types of hearing aids. Their primary purpose is to improve the hearing of sounds by amplifying incoming signals. The US Food and Drug Administration (FDA) states that the hearing aid should be considered as a personal sound amplification product (PSAP) [4]. Recently, PSAPs have appeared as OTC devices on the market. They are much cheaper than classic hearing aids but less efficient especially in patients with moderately severe hearing loss [5]. Using PSAPs, the gain was insufficient, especially for high frequencies. Although interest in PSAPs has increased globally, most people who choose hearing aids are HAs through hearing care professionals, even though they can purchase OTC devices [5]. Well-chosen PSAPs can be helpful in communication in everyday life [5]. Nevertheless, the required first line of the diagnosis and treatment of hearing loss is consultation with an audiologist.

It is estimated that although more than 400 million people worldwide would benefit from hearing aids [3], unfortunately, only 17% of them (68 million) use hearing aids [6]. These data depend on the age group. In the CONSTANCES among participants aged 18 to 25 years, using hearing aids was reported by 56.7%; in the group aged 65 to 70 years, it was 36.0%; and among participants aged 71 to 75 years, it was only 32.9% [7].

The importance of the aspect of improving hearing through the use of hearing aids is evidenced by the MarkeTrak research organized in the United States since 1989, which allows us to analyze not only the needs of the patients but also the problems reported by the patients. Following the example of the MarkeTrak research, EuroTrak research has been organized in Europe since 2011 [8]. They found that the overall satisfaction of Europeans with hearing aids is relatively high (72% to 86% depending on the country) and comparable to the North Americans (74%) [8,9], while 96% of hearing aid owners declare that their hearing aids at least sometimes improve their quality of life [10].

An essential element for the best fitting of hearing aids is still an interview with the patient (anamnesis), information about the sound environment in which the patient lives, assessment of the degree of intelligence and manual skills, evaluation of the patient’s expectations and audiometric measurements and audiological tests of the hearing organ.

The audiometric tests are necessary in order to qualify for hearing aids and include pure tone audiometry—determination of the hearing threshold curve as a function of frequency (HTL—hearing threshold level), determination of the level of comfortable hearing (MCL—most comfortable loudness level), the level of discomfort of hearing (UCL—uncomfortable loudness level) or speech intelligibility testing in a free field.

Particularly noteworthy is the result of pure tone threshold audiometry. It allows the patient to be pre-qualified for hearing prostheses and, above all, to be qualified by the state or other organizations for a full refund or partial refund of the hearing aid costs. Extending reimbursement to two hearing aids is a very important step that enables patients to maximize the benefits of their hearing aids [11].

The aim of this study is to compare audiometric results and demographic structure in patients qualified for hearing aids in two different periods (1996–2001 and 2016–2021).

## 2. Materials and Methods

The material covered 1246 patients of the Audiological Clinic of the Mazovian Bródno Hospital randomly selected from among people qualified for hearing aids with air and bone conduction devices in the years 1996–2001 and 2016–2021. The patients were briefed information about the research, and those who agreed to participate signed informed consent before the study started. The patients ranged in age from 18 to 95 (mean 70, SD = 12.5), and the qualifications were performed by the same medical team in each case. Pure tone threshold audiometry was performed on each patient included in the study. Additionally, from patients who qualified in the years 2016–2021, data on the type of hearing aid they chose during the qualification were collected.

Hearing-impaired patients were divided into two groups according to the time of qualifying for hearing aids. Group 1 consisted of 759 people who qualified for hearing aids in the years 1996–2001, and Group 2 consisted of 487 people who qualified in the years 2016–2021.

Statistical analysis was performed on the results of pure tone threshold audiometry, which were the basis for qualification for hearing aids, and the demographic structure in both groups. Descriptive statistics and testing of hypotheses using the parametric repeated-measures Student’s *t*-test were used. The statistical tests were performed in R statistical software.

## 3. Results

**Figure 1:**
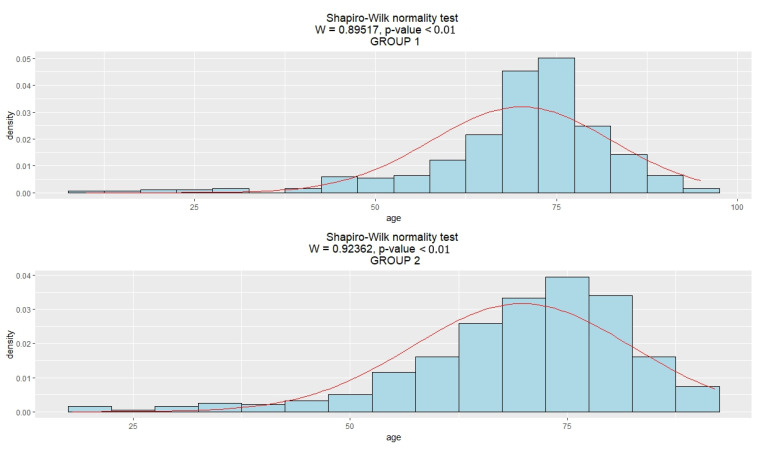
Histogram graphs (normal distribution) for age in patients in the study groups: Group 1 (1996–2001); Group 2 (2016–2021). Red line—expected norm.

The analysis of the demographic structure of the patients in both groups using the Shapiro–Wilk test did not show statistically significant differences in the age of people qualified for hearing aids over the two periods of observation (Figure 1).

**Figure 2:**
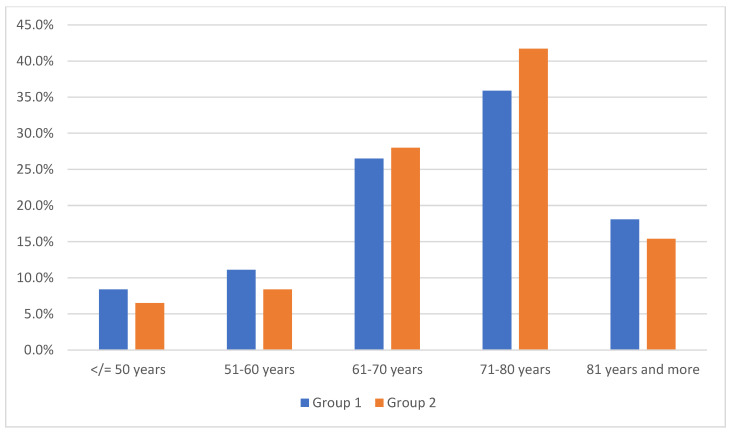
The basic statistical data of individual age groups.

In both groups, more than half of the people qualified for hearing aids were over 70 years of age, and the largest population were patients in the 71–80 age group (Figure 2).

However, the demographic structure of patients covered by hearing aids has changed in terms of gender distribution. Twenty-five years ago, hearing aids were used significantly more often by men, and they constituted about 60% of the group. Currently, the ratio of women to men has evened out and even showed a slight advantage of women over men (53% vs. 47%) (Table 1).

Statistical analysis of audiometric test results showed no statistically significant differences in the average hearing threshold (dBHL) between the right and left ear within particular groups (Table 2).

The patients were divided into four groups in terms of grades of hearing loss as follows: mild (21–40 dB), moderate (41–60 dB), severe (61–80 dB) and profound (≥81 dB). Nearly 50% of patients in Group 2 had mild or moderate hearing loss, which is a significantly higher percentage than in Group 1 (Table 3).

**Figure 3:**
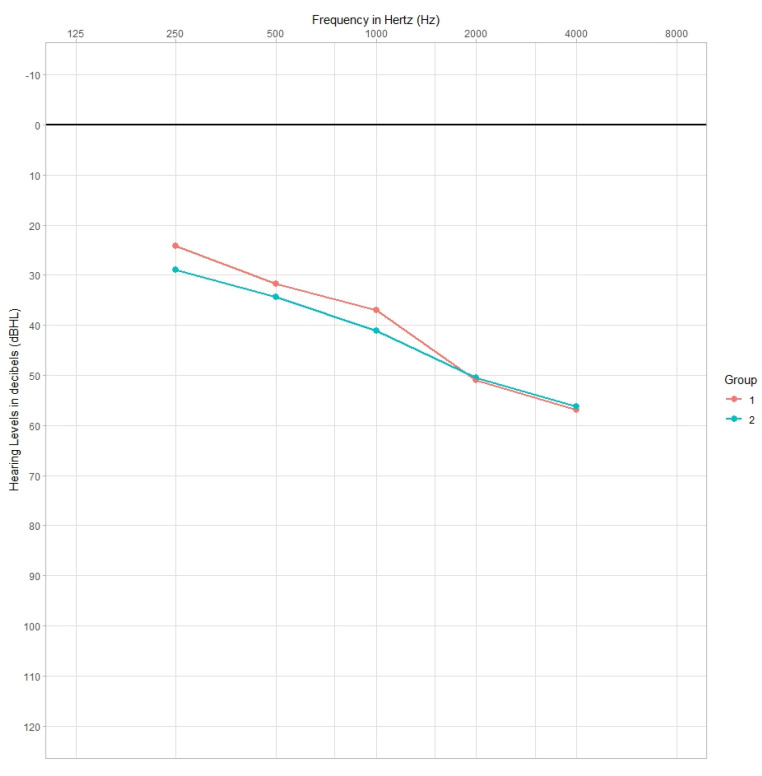
Mean bone conduction hearing thresholds (dBHL) of patients in Group 1 and Group 2.

**Figure 4:**
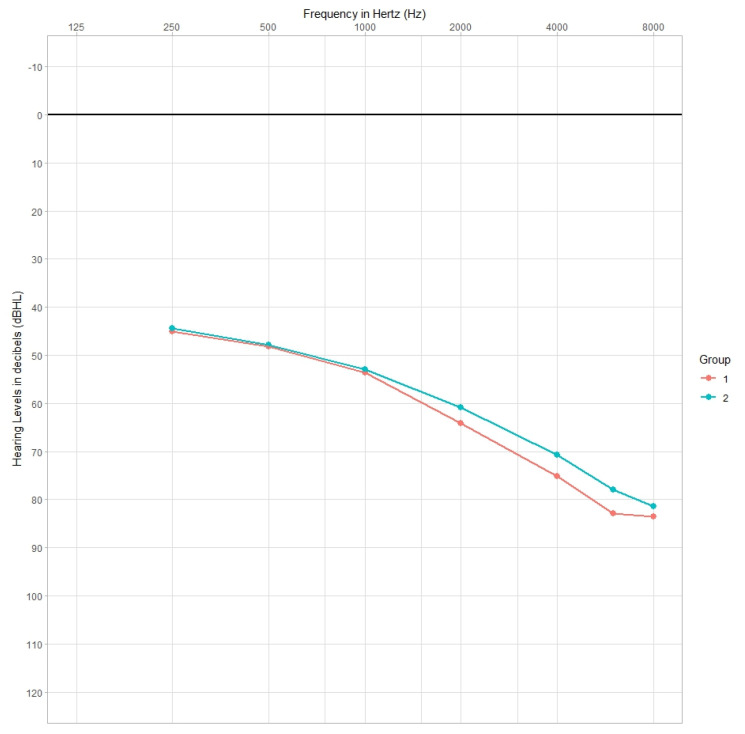
Mean air conduction hearing thresholds for patients in Group 1 and Group 2.

The comparison of the mean hearing thresholds between group 1 and group 2 for both bone conduction (Figure 3) and air conduction (Figure 4) showed statistically significant differences between the groups.

On the basis of statistical analysis (Student’s *t*-test), it was found that patients from Group 1 had significantly elevated air conduction hearing thresholds for sounds with frequencies from 2000 to 8000 Hz compared to patients from Group 2. This difference ranged from 2 to 5 dB and was highest at 4000 and 6000 Hz. Simultaneously, it was observed that Group 2 patients had significantly elevated bone conduction hearing thresholds at frequencies from 250 to 1000 Hz relative to Group 1 patients, and this difference ranged from 2 to 5 dB and was highest at 250 Hz (Table 4, Figure 3 and Figure 4).

Additionally, from the patients in Group 2, the information on the types of hearing aids they chose during the qualification process was collected. The type of hearing aid used by Group 2 was analyzed and presented in Table 5. The most popular type was the behind-the-ear hearing aid (BTE), chosen by 84% of patients. In the group of patients under 60 years old, in-the-canal hearing aid (ITC) was significantly more popular at 28.6% than in patients aged 60 and over, where the ITC accounted for only 13.4%. Patients with binaural hearing aids accounted for 13.3% of all patients.

## 4. Discussion

The development of medical technology has led to progress in the field of hearing aids in recent years. It allows patients to use devices that are more efficient, easier to use and improve the quality of hearing more effectively. Recently, smartphone-connected hearing aids have been more popular [12]. Patients, who used smartphone-connected hearing aids in their everyday lives, were viewed positively by participants across a range of domains, empowering them and enabling hearing loss self-management [12].

The relationship between hearing loss and age is so strong that it can be concluded that almost everyone, if they live long enough, will have some degree of hearing loss, and at least 50% will have moderate to complete hearing loss [1]. Because the world’s population is growing and aging in the coming decades, the demand for hearing aids will increase.

People are more and more willing to use hearing aids and decide to use them earlier than in the past. This is due, among others, to greater social awareness of the effects of hearing loss and the possibility of preventing hearing deterioration through the use of prostheses. Attitudes towards the use of hearing aids are also changing. Modern models are more aesthetic and discreet. Among adults over the age of 70, the proportion of people who reported owning and using hearing aids increased by 23.3% between 2011 and 2018 in the US [13]. However, the overall trend of increasing hearing aid use masks significant differences by race and gender [13]. Significantly, participants with lower incomes experienced a proportional decrease in the ownership and use of hearing aids [13]. Therefore, the role of the health policy regarding the reimbursement of hearing aids and the availability of otolaryngologists and audiologists is crucial.

The early detection of hearing loss and implementation of appropriate hearing aids can decrease the risk of developing depression, particularly in people of a lower socioeconomic status and in older adults [14,15]. Hearing aid fitting is more difficult in the case of a long history of untreated hearing loss [16]. The assumption here is that the ability to perceive and process speech in the brain is progressively reduced in the absence of stimulation [17]. Regular use of hearing aids may also have a protective effect on cognitive functions in those patients with moderate hearing loss [18]. Hearing aids may increase social activity level and decrease social participation restriction [19].

The individual approach to the patient is an important aspect of hearing loss treatment. Gender may elicit different responses from one’s hearing care professional. Consequently, the diagnosis and treatment of hearing loss may vary according to gender [20], although this topic remains to be investigated specifically in hearing health care. Recently, interesting research has been carried out, among others, on dealing with hearing loss and adaptation to wearing hearing aids in relation to gender [21]. Worse adaptation to hearing aids was observed among men who reported a less positive attitude towards hearing aids. Women are better at adapting to wearing hearing aids [21].

Our research suggests that more and more women are choosing to wear hearing aids. Twenty-five years ago, they accounted for only 40% of prosthesis patients, and today, they are the dominant gender (53%). This may be related to the fact that women live longer than men and are more likely to suffer from age-related hearing problems. In addition, modern women have less visible appliances at their disposal, which reduces their aesthetic concerns. Changing the structure of employment in society has also meant that women need to use hearing aids at work more often than they used them when working at home, which probably makes them more inclined to use hearing aids with prostheses.

The main goal of each method of fitting hearing prostheses is to select the device that corrects the hearing defect and to set its characteristics to improve the hearing comfort of the hearing-impaired person in the best possible way and in particular to improve the level of speech intelligibility.

According to the accepted standards, an adult patient (over 26 years of age) qualifies for hearing aids with bilateral hearing loss on average above 40 dB for frequencies corresponding to the speech range. It should be noted, however, that this is an “administrative” criterion and does not take into account so-called “steep” (“high-frequency”) hearing losses. It also does not take into account the lack of intelligibility of colloquial speech even with smaller hearing losses for pure tones. That is why the fitting process itself is so important. Especially patients with sensorineural hearing loss have difficulty with understanding speech even with carefully selected parameters of hearing aids. In 2022, the prospective study proved that hearing rehabilitation therapy significantly improves speech understanding in hearing aids in patients with sensorineural hearing loss [22].

According to the National Institute of Deafness and Other Communication Disorders (NIDCD), approximately 28.8 million adults in the United States may benefit from wearing hearing aids. In 2016, 3.65 million hearing aids were issued in the United States, and the average age of first-time hearing aid wearers was 70. It was also found that a significant number of people waited up to 6 years from the time they first noticed their hearing loss to purchasing their first hearing aid [23].

In our study, the patients in the years 1999–2001 (Group 1) made the decision to use hearing aids at more advanced stages of hearing loss than similar decisions the patients made today (Group 2). This statement is supported by the fact that patients with mild or moderate hearing loss constituted 49.9% of the whole Group 2, which is significantly higher than in Group 1 at 42.8%. Moreover, the hearing threshold for air conduction in the frequency range from 2000 to 8000 Hz was 2–5 dB elevated in Group 1 than in Group 2. This is especially important due to the fact that these frequencies are responsible for “the speech understanding”. These results are in line with the latest population studies, which show that more and more patients with hearing loss decide to use hearing aids. In the case of patients with moderate hearing loss (40–59 dB), a significant increase in the prevalence of the use of hearing aids in the group of patients from 2011 to 2016 was found, compared to the study group between 1999 and 2004 [13,24].

At the same time, patients from Group 2 had significantly higher hearing thresholds in the bone conduction range at frequencies from 250 to 1000 Hz compared to patients from Group 1, which resulted in greater difficulties with the selection of hearing aids.

Similar results were obtained in Poland in 2016 based on the EuroTrak study, where people over 74 were dominant among people with prostheses—48.4% [11]. No tendency to change the age structure of people with prostheses in recent years was also observed in studies conducted in the United States, comparing the years 2005–2006 and 2010–2011. Slightly different data were obtained by Reed et al., who found an increase in the percentage of people wearing hearing aids aged 70 and older from 15.0% in 2011 to 18.5% in 2018 [13]. With the increase in age, the percentage of elderly people with hearing aids also increased [13].

Contrary to our observations, American scientists did not notice changes in the sex structure of the prosthetic people, which we noted in our research [4]. This difference may result from a different period analyzed as well as from different cultural features of both societies.

In our study, in Group 2, we also analyzed the types of hearing aids chosen by patients. Among them, classic behind-the-ear (BTE) hearing aids were the most popular—84%. In patients under 60 years of age, in-the-canal devices (ITC) accounted for 28.6%, while in the group aged 60 years and over, they accounted for only 13.4%. The lower popularity of ITCs in elder patients may be due to the fact that BTEs are easier to use, have larger batteries, are harder to lose and, perhaps crucially, have a lower price compared to ITCs. However, the greater popularity of ITC in younger people may be due to the aesthetic qualities of this type of hearing aid, and above all, they are less visible.

Importantly, apart from aesthetic considerations, no statistically significant differences were observed in the effectiveness of Lyric Extended Wear (EW), behind-the-ear open hearing aids (RITE) and fully in-the-canal (CIC) hearing aids [25].

It is noteworthy that only 13.3% of our patients used binaural hearing aids. This percentage is much lower compared to the results of the Eurotrak study, where in 2009 in Germany, 60% of patients using hearing aids had hearing aids in both ears, and in the USA in 2008, 74% of patients used hearing aids in both ears [26]. This may be due to the fact that the extension of the reimbursement of hearing aids for both ears came into force in Poland in 2014. Despite the refund, the economic aspect probably has a large share in such a low prevalence of hearing aids for both ears. An important task for audiologists, laryngologists and general practitioners is to disseminate knowledge among patients that binaural hearing aids have significant advantages. They significantly increase speech understanding, enable correct sound localization and stimulate bilaterally the hair cells for the conduction of stimuli. In the research by Xu Wu et al., patients with presbycusis had higher satisfaction using bilateral hearing aids than unilateral ones [27].

At the same time, it should be emphasized that as many as 96% of hearing aid owners declared that their hearing aids at least sometimes improve their quality of life. Hearing aid users are 14.5% less exhausted at the end of the day compared to non-hearing aid users with similar hearing loss and are less likely to experience depression or memory loss [11]. It has been proven that the use of hearing aids stimulates the plasticity of the brain, and after 4 weeks, the records in the cortical auditory evoked potentials’ change [28,29]. Moreover, for patients with moderate hearing loss, the use of hearing aids has a suppressive effect on cognitive impairment [18].

## 5. Conclusions

1. Patients who decided to wear hearing aids in 1999–2001 (Group 1) had significantly higher hearing thresholds in the air conduction range at frequencies from 2000 to 8000 Hz in relation to patients with modern prostheses (Group 2).
2. Patients nowadays (Group 2) decide on hearing aids at an earlier stage of hearing impairment compared with patients in Group 1.
3. Patients in Group 2 had significantly higher hearing thresholds in the bone conduction range at frequencies from 250 to 1000 Hz compared to patients in Group 1, which may cause greater difficulties with the selection of hearing aids.
4. The age structure of patients qualified for hearing aids has remained at a similar level over the last 25 years. On the other hand, the gender structure of people assisted by hearing aids has changed significantly.

## Figures and Tables

**Table 1:** Demographic structure of both study groups.

Gender	GROUP 1		GROUP 2	
	1996–2001		2016–2021	
	Count	Mean Age	Count	Mean Age
Female	309 (40.7%)	69.4	257 (52.7%)	69.5
Male	450 (59.3%)	70.8	230 (47.3%)	70.1
Total	759	70.2	487	69.9

**Table 2:** Comparison of average hearing thresholds (dBHL) between the right and left ear in particular study groups.

Group	Type of Conduction	f (Hz)	EL	ER	t	*p*
GROUP 1	Air conduction	250	44.93	44.14	−0.11	0.91
		500	48.25	48.05	0.19	0.85
		1000	53.88	53.33	0.50	0.62
		2000	64.30	61.64	0.33	0.74
		4000	75.40	70.89	0.33	0.74
		6000	82.71	78.07	−0.34	0.73
		8000	83.47	81.24	−0.20	0.84
	Bone conduction	250	24.53	29.25	0.68	0.50
		500	31.69	34.52	−0.21	0.84
		1000	37.03	41.50	0.20	0.84
		2000	50.81	50.75	−0.35	0.73
		4000	57.01	56.22	0.14	0.89
GROUP 2	Air conduction	250	45.06	44.72	−0.40	0.69
		500	48.03	47.77	0.19	0.85
		1000	53.35	52.70	0.44	0.66
		2000	63.95	60.08	1.13	0.26
		4000	75.04	70.52	0.27	0.79
		6000	83.08	77.71	0.26	0.80
		8000	83.65	81.75	−0.34	0.73
	Bone conduction	250	23.99	28.51	0.81	0.42
		500	31.88	34.22	0.30	0.76
		1000	36.84	40.75	0.69	0.49
		2000	51.14	50.14	0.57	0.57
		4000	56.88	56.34	−0.12	0.91

Legend: ER—right ear, EL—left ear.

**Table 3:** Grades of hearing loss among study groups.

Hearing Loss	Group 1	Group 2
mild	5.5%	2.9%
moderate	37.3%	47.0%
severe	35.8%	33.1%
profound	21.4%	17.0%

**Table 4:** Comparison of average hearing thresholds (dBHL) for bone and air conduction between Group 1 and Group 2 for each frequency.

Site	Type of Conduction	f (Hz)	Group 1	Group 2	t	*p*
EL	Air conduction	250	44.93	44.14	0.63	0.53
		500	48.25	48.05	0.15	0.88
		1000	53.88	53.33	0.45	0.65
		2000	64.30	61.64	2.24	0.03
		4000	75.40	70.89	3.72	0.00
		6000	82.71	78.07	3.81	0.00
		8000	83.47	81.24	1.90	0.06
	Bone conduction	250	24.53	29.25	−5.28	0.00
		500	31.69	34.52	−2.79	0.01
		1000	37.03	41.50	−4.28	0.00
		2000	50.81	50.75	0.06	0.95
		4000	57.01	56.22	0.80	0.42
ER	Air conduction	250	45.06	44.72	0.26	0.79
		500	48.03	47.77	0.20	0.84
		1000	53.35	52.70	0.52	0.61
		2000	63.95	60.08	3.20	0.00
		4000	75.04	70.52	3.60	0.00
		6000	83.08	77.71	4.27	0.00
		8000	83.65	81.75	1.58	0.01
	Bone conduction	250	23.99	28.51	−5.43	0.00
		500	31.88	34.22	−2.41	0.02
		1000	36.84	40.75	−3.91	0.00
		2000	51.14	50.14	0.95	0.34
		4000	56.88	56.34	0.53	0.60

Legend: ER—right ear, EL—left ear.

**Table 5:** Type of hearing aids in Group 2.

Type of Hearing Aids	Total	ER	EL	Both Sides
In the canal (ITC)	78 (16.0%)	34	24	20
Behind the ear (BTE)	409 (84.0%)	205	159	45

## Data Availability

Data available on request due to restrictions privacy. The data presented in this study are available on request from the corresponding author.
